# Root canal reconstruction using biological dentin posts: A 3D finite element analysis

**DOI:** 10.15171/joddd.2019.042

**Published:** 2019

**Authors:** Seda Falakaloğlu, Özkan Adıgüzel, Gökhan Özdemir

**Affiliations:** ^1^Department of Endodontics, Faculty of Dentistry, Afyonkarahisar Health Sciences University, Afyonkarahisar, Turkey; ^2^Department of Endodontics, Faculty of Dentistry, Dicle University, Diyarbakır, Turkey; ^3^Dental Prosthetics Technology, Vocational School of Health Services, Bahçeşehir University, Istanbul, Turkey

**Keywords:** Biomimetic, dentin post, finite element analysis

## Abstract

***Background.*** Several types of post have been developed for clinical use. A biological dentin post obtained from an extracted tooth eliminates the problems arising from material differences and reduces the fracture rate in teeth undergoing root canal treatment. This study used finite element analysis to compare a biological dentin post with posts made of two different materials.

***Methods.*** Three 3D models of the upper central incisor were created, and stainless-steel, glass fiber and biological dentin posts were applied to these models. The restoration of the models was completed by applying a composite as the core structure and a ceramic crown as the superstructure. Using finite element stress analysis in the restoration models, a 100-N force was applied in the vertical and horizontal directions and at a 45º angle, and the suitability of the biological dentin post was evaluated by comparing the data.

***Results.*** Under the applied forces, the greatest stress accumulation was seen in the models with the stainless steel post. Because the stainless steel post was more rigid, stress forces accumulated on the surface instead of being transmitted to the tooth tissue. In the models with the glass fiber and biological dentin posts, the post material responded to the stratification in tandem with the dental tissue and did not cause excessive stress accumulation on the tooth or post surfaces.

***Conclusion.*** The results showed that biological dentin posts prevent the accumulation of stresses that might cause fractures in teeth undergoing root canal treatment. In addition, the physical compatibility and biocompatibility of a biological dentin post with the tooth imply that it is a good alternative to the types of post currently used.

## Introduction

Many procedural factors contribute to the success of endodontic treatment. The most important factor in the success of root canal treatment is the restoration applied.^1^ Therefore, the restoration of endodontically treated teeth is currently among the most frequently discussed topics. Teeth that have undergone excessive dentin loss should receive post-core restorations. The amount of stress after the application of endodontic treatment to teeth differs among post material, periodontal ligament, and bone. This is because stress fractures can occur on the surfaces of the tooth and the post. The post material and its properties play a critical role in the process of tooth fracture, which is rehabilitated by post-core application.^2^

The physical properties (e.g., elastic modulus) of the post material must be similar to those of dentin; moreover, the post must be connected to the structure of the tooth and must be biocompatible in the oral environment. Among the post materials used, fiber posts have the closest elastic modulus to dentin. Following fiber post application, post-core applied teeth have been shown to accumulate stress, especially in the cervical area. There is no post material with properties better than those of a fiber post.^3^

Finite element stress analysis is a numerical modeling method developed to solve various complex problems in engineering. It can also be used in biomechanical studies to assess engineering-related aspects. Notably, this method has been successfully applied for stress analysis in various areas of dentistry.^4^ The aim of this study was to compare stress resistance between stainless steel posts, glass fiber posts, and biological dentin posts in the restoration of endodontically treated teeth in a three-dimensional virtual environment, which provides ideal conditions for observations that cannot be performed in the oral cavity. The data obtained in this study can be used to evaluate the applicability of biological dentin in the clinic.

## Methods

In this study, an upper central incisor tooth was selected for finite element stress analysis. A three-dimensional solid model was obtained using Rhinoceros 4.0 software (Robert McNeel & Associates, Miami, FL, USA) set to the average size indicated in Wheeler's Dental Form Atlas (a total tooth length of 22 mm, a root length of 14 mm).^5^ The root canal filling was designed within the model. To ensure apical sealing, a 4-mm gutta-percha model was adapted to the root canal system with a length 1 mm shorter than the tooth apex. The solid model and environmental textures obtained were modeled with a mesh containing 15,270 elements, each of which had elastic properties and connected with 31,327 nodes, representing the material being modeled. The posts used in the study measured 1.2 mm in diameter and 14 mm in length. All the posts were designed with a cylindrical structure.^6^ The post segment remaining in the root measured 10 mm and a 4-mm portion was in the structure of the core. The mechanical properties of the composite resins (Filtek Supreme XT, 3M ESPE, St. Paul, MN, USA) were used for the restoration of the core. Assuming that the tooth would be restored with a full-ceramic crown (IPS Impress, Ivoclar/Vivadent, Liechtenstein, Schaan), 1-mm-thick chamfer-designed steps were created around the tooth; these steps ended at the gingiva level. The amount of occlusal reduction in the prepared tooth form was 2 mm, and the amount of axial reduction was 1 mm. The angles of the axial walls were 6–8°.^7^ Zinc phosphate cement was used between the stainless steel post and the tooth, whereas the glass fiber and biological dentin posts were applied using a resin cement (Clearfil SA Cement Kuraray Co., Tokyo, Japan). In addition, a resin cement was used between the ceramic crown and the core material. The thickness of the cement was determined to be 0.1 mm. The forces from the three different directions to each post model were 100 N. F_1_, vertical force: force applied parallel to the long axis of the tooth (0°); F_2_, chewing force: force at a 45° angle to the long axis of the tooth applied from the palatal aspect; F_3_, horizontal force: from the labial aspect to the long axis of the tooth (90°).

In ANSYS 14 software (Ansys, Inc., Houston, TX, USA), it is necessary to introduce the properties of the structures to be constructed. Therefore, the structures were assigned to the material mechanical properties (elastic modulus and Poisson ratio) that defined their physical characteristics. The elastic properties of the isotropic materials are reported in Table 1.

**Table 1:** Properties of the materials

**Material**	**Elastic modulus (E) Gpa**	**Poisson’s ratio** **(V)**	**References**
**Cancellous bone**	1.37	0.30	8
**Compact bone**	13.7	0.30	8
**Dentin**	18.6	0.31	8
**Cement**	18.6	0.31	9
**Periodontal ligament**	0.0689	0.45	8
**Gingiva**	0.003	0.45	10
**Gutta-percha**	0.00069	0.45	8
**Stainless-steel post (ParaPost, Coltène/ Whaledent Inc., USA)**	206	0.33	Provided by manufacturer
**Glass-fiber post (Snowlight, Carbotech, USA)**	49	0.28	Provided by manufacturer
**Ceramic crown**	96	0.3	11
**Composite core (Filtek Supreme XT, 3M ESPE, USA)**	10.4	0.27	Provided by manufacturer
**Adhesive cement**	2.8	0.33	12
**Zinc phosphate cement**	22.4	0.25	13

## Results

Under all the forces, the concentration of stress at the force application area is expected to be higher than the initial force. The stress values obtained from stress analysis represent the maximum von Mises stress values obtained from the models. The mathematical values are given in MPa. The stress values of F_1_, F_2_ and F_3_ are shown in Table 2.

**Table 2:** Stress values of F_1_, F_2_ and F_3_ forces applied to the models

**Force direction**	**Post type**	**Maximum von Mises stress values (MPa)**	**Minimum von Mises stress values (MPa)**
**F**_1_ **(vertical force)**	Stainless steel post	70.021	0.000086520
	Glass fiber post	70.367	0.000081250
	Biological dentin post	70.747	0.000074412
**F**_2_ **(masticatory force)**	Stainless steel post	44.187	0.0000621900
	Glass fiber post	44.54	0.0000626444
	Biological dentin post	44.83	0.0000631720
**F**_3_ **(horizontal force)**	Stainless steel post	8.4189	0.0000037919
	Glass fiber post	8.5898	0.0000038291
	Biological dentin post	8.7033	0.0000035987

In the models where the F_1_ force was applied from the incisal edge of the tooth at 0° parallelism to the long axis, the stress was concentrated on the core and on the post. In particular, the amount of stress accumulated during the procedure was maximum in the stainless steel post model. The distributions of stress in the biological dentin and glass-fiber post models were similar. Less stress accumulated on the root surface than on the surfaces of the crown and post.

In the models with force applied at a 45° angle to the palatal surface of the tooth, and in which the F_2_ force was applied to mimic a masticatory force, stress accumulated in the area where the force was applied. More stress accumulated on the root surface of the palatal area than in the labial direction. In particular, the stainless steel post exhibited more stress than the other posts. The distributions of stress in the glass fiber and biological dentin posts were similar.

**Figure 1:**
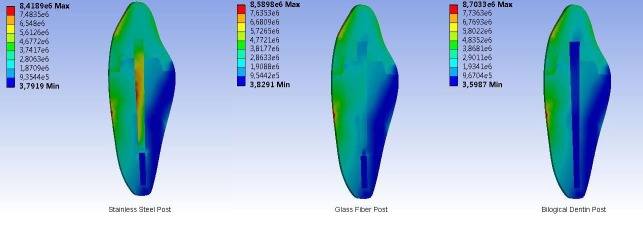
Stress distribution under F_3_ force (e6 = MPa).

In the models of the labial surface of the tooth with a horizontal F_3_ force applied at a 90° angle, the area where the force was applied and the amount of stress accumulated on the root surface in the same direction were significantly higher than the areas and stress amounts for other models (Figure 1). All the models showed increased stress accumulation in a particular area in the middle third of the crown and root. In comparison to the amount of stress that accumulated on the surface of the post, the stress distribution of the biological dentin post was more similar to that of the root dentin in the palatal region, while the stress was higher in the stainless steel post. The stress was lower in the glass-fiber post than in the stainless steel post but was higher than the stress in the root dentin.

**Figure 2:**
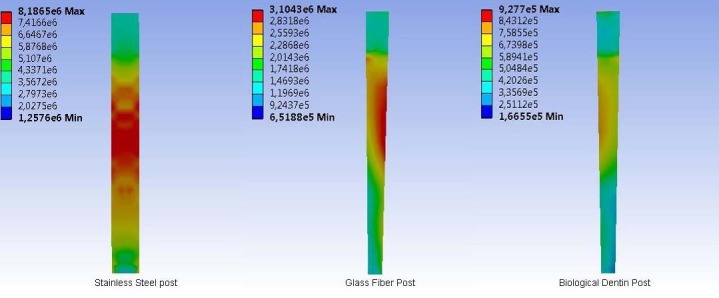
Stress distribution under F3 force in posts (e6 = MPa).

Stress in the post materials was evaluated upon the application of F_1_, F_2_ and F_3_ forces; the resultant values are shown in Table 3. Significant differences were found between the three post systems; the highest stress values were recorded in the stainless steel posts. When stress distributions occurred on the post surfaces, no homogenous distribution was observed in any post. In particular, stress accumulation was the highest in stainless steel and glass fiber posts when the F_3_ force was applied; moreover, stress accumulation occurred in the middle region of the posts (Figure 2). Stress accumulation in the biological dentin post was comparatively small. As the modulus of elasticity increased, the stress increased in the internal structure of the post material. The accumulation of stress in the inner structure of the stainless steel post resulted in the transmission of more stress to the root dentin.

**Table 3:** Stress values of F_1_, F_2_, and F_3_ forces applied to the posts

**Force direction**	**Post Type**	**Maximum von Mises stress values (MPa)**	**Minimum von Mises stress values (MPa)**
**F**_1_ **(vertical force)**	Stainless steel post	22.77	2.15070
	Glass fiber post	8.6574	0.72826
	Biological dentin post	3.623	0.37500
**F**_2_ **(masticatory force)**	Stainless steel post	19.369	2.18940
	Glass fiber post	7.0366	1.25030
	Biological dentin post	2.0995	0.38162
**F**_3_ **(horizontal force)**	Stainless steel post	8.1865	1.2576
	Glass fiber post	3.1043	0.65188
	Biological dentin post	0.9277	0.16655

## Discussion

An ideal post system should have the following characteristics: It should not create stress in the teeth or any related fractures, should be easy to apply, and should not require excessive loss of material from the tooth.^14^ Fiber posts are most widely accepted by researchers and have an elastic modulus which is very similar to that of dentin.^15^ It has been reported that a material similar to dentin in hardness reduces the amount of stress and causes fewer root fractures.^12^ The ideal post material should exhibit appropriate elastic modulus, compressive strength and thermal expansion coefficient, in addition to fulfilling aesthetic requirements.^16^ Based on these properties, researchers have used dentin as a post material in recent years and have achieved successful clinical results.^17,18^ Therefore, we evaluated the formation of stress during the use of biological dentin posts.

In this study, finite element stress analysis was used to evaluate stress in endodontically treated teeth. Many studies have shown that this is the ideal method for assessing post-core application, compared to several other methods of stress analysis.^19-21^ With other methods, the area of analysis is limited, whereas finite element stress analysis allows assessment of the distribution and amounts of stress in each area of the structure.^22^ The directions and degrees of force applied can be compared; depending on the force, relevant changes can be observed.^23^ In a study of the reasons for the failure of post-core systems due to material fatigue, finite element stress analysis revealed that modeling standardization was important to ensure reliable values representative of the stress experienced by the teeth.^24^

Escribano et al^2^ used finite element stress analysis to compare stainless steel and glass fiber posts, and found that the glass-fiber post transmitted less stress to the tooth than did the stainless steel post; moreover, stress in the stainless steel post accumulated primarily on the post surface, around the post, and in the structures of dentin and cement. Therefore, stainless steel posts were presumed to give rise to irreparable tooth fractures. The findings of the present study support this theory. Compared to the stainless steel post, stress accumulation on the glass fiber post surface was very low. The physical properties of the material favor the glass fiber post because its modulus of elasticity is similar to that of dentin. When force was applied in three different directions in our model, stress accumulation was greater on the surface of the post in all the cases. Therefore, to increase the success of post‒coronal restorations in the clinic, it is important to use post materials that have physical properties similar to those of dentin, rather than metal posts.

In the models used for our analysis, the numbers of elements and nodes were 15,270 and 31,327, respectively. Sorrentino et al^21^ used 13,272 elements and 15,152 nodes; Belli et al^25^ used 34,515 elements and 13,300 nodes; and Lanza et al^26^ used 13,272 elements and 15,152 nodes. Thus, the models in the present study were prepared with characteristics similar to those used in previous studies.

Horizontal and vertical forces represent the forces from situations such as falling, traumatic forces, and bending in the centric occlusion.^8,27,28^ Helkimo et al^29^ reported that during chewing, the amount of force applied to the incisor teeth varied between 100 N and 200 N. The force used in our study was set at 100 N, which is within the limits of masticatory forces, and was then applied in three different directions. When we compared the stress values of the forces applied to the models created in our study along the crown-root, we found that the highest values were formed when horizontal forces were applied, followed by vertical forces and chewing forces. Amarnath et al^30^ reported that horizontal forces were the most stressful in the oral cavity. When horizontal forces were applied, in the models with stainless steel and glass fiber posts, there was considerable stress accumulation on the post surface; in contrast, in the biological dentin post, similar stress accumulation was observed on the palatal surface of the root because the biological dentin post exhibited a modulus of elasticity similar to that of dentin. The module is equipped with a rigid structure and high elasticity because it is more resistant to bending in the face of the forces concentrated on the structure.^31^ Although this situation was observed in other forms of posts, it was not observed in the biological dentin post. Gloria et al^32^ stated that stress distribution was along the post and at the interface between the post and the surrounding structure. In a similar study by Belli et al,^25^ a 300-N force was applied to the palatal region; the resultant stress was concentrated in the cervical region of the models. In this study, the amount of stress increased in the middle of the palatal and labial root surfaces in models with chewing forces, due to full-ceramic crown modeling and the application of reduced force. When teeth are restored, planning should be performed with an emphasis on the remaining tooth structure and functional factors; the load received depends on the tooth position in the arch, as well as occlusion and rehabilitation planning.^33^ These aspects should also be considered in future studies on biological dentin posts.

There are few studies in the literature regarding biological dentin posts.^17,25,34-36^ Correa-Feria et al^17^ reported that this post treatment, defined as biological restoration by Trope,^37^ is a successful alternative to reconstruction in endodontically treated teeth. A dentin post study by Belli et al^25^ used a methodology similar to that of our study; however, in the present study, a complete ceramic crown was applied to the post-applied models, whereas the force was directed to the cervical region in the study by Belli et al.^25^ Therefore, stress accumulation was observed only in regions where the force was applied, while greater stress was observed in the stainless steel post. The lowest stress value was found in the model that used a biological dentin post. Therefore, the results of these biological dentin post studies using finite element stress analysis are inconsistent. However, in both the model and in biological dentin posts, the isotropic structure is a notable limitation of the finite element stress analysis. The results obtained from stress analysis of finite elements in different models and the fracture strength tests on the extracted teeth will increase our knowledge of biological dentin posts.

## Conclusions

Within the limitations of this study, the results imply that biological dentin can be used as a post material under static and fatigue loading. Biological dentin posts are a viable alternative to traditional materials. However, there should be more studies on biological dentin posts. Non-homogeneous stress distribution was observed in the glass fiber post, whereas less stress was distributed homogeneously in the model of a biological dentin post.

## Competing Interests

The authors declare that they have no competing interests.

## Authors’ Contributions

SF and OA contributed to the concept and the design of the study. SF performed the experiments. SF and GO drafted the manuscript. All the authors contributed to the critical revision of the manuscript, and have read and approved the final paper.

## Acknowledgments

This work was supported by Dicle University Scientific Research Projects Coordinatorship (Project no: DIS.16.016).

## Funding

Not applicable.

## Ethics Approval

Not applicable.
